# Design principles, manufacturing and evaluation techniques of custom dynamic ankle-foot orthoses: a review study

**DOI:** 10.1186/s13047-022-00547-2

**Published:** 2022-05-19

**Authors:** Giulia Rogati, Paolo Caravaggi, Alberto Leardini

**Affiliations:** Movement Analysis Laboratory, IRCCS Istituto Ortopedico Rizzoli, Via di Barbiano 1/10, 40136 Bologna, Italy

**Keywords:** Ankle foot orthosis, Dynamic, Custom, Drop-foot, Additive manufacturing, 3D scanning, Functional evaluation, PD-AFO, Comfort, design

## Abstract

Ankle-Foot Orthoses (AFO) can be prescribed to allow drop-foot patients to restore a quasi-normal gait pattern. Standard off-the-shelf AFOs are cost-effective solutions to treat most patients with foot and ankle weakness, but these devices have several limitations, especially in terms of comfort. Therefore, custom AFOs are increasingly adopted to address drop-foot when standard solutions are not adequate. While the solid ones are the most common type of AFO, providing full stability and strong resistance to ankle plantarflexion, passive dynamic AFOs (PD-AFOs) represent the ideal solution for patients with less severe ankle weakness. PD-AFOs have a flexible calf shell, which can bend during the stance phase of walking and absorb energy that can be released to support the limb in the push-off phase. The aim of this review is to assess the state-of-the-art and identify the current limitations of PD-AFOs. An extensive literature review was performed in Google Scholar to identify all studies on custom PD-AFOs. Only those papers reporting on custom PD-AFOs were included in the review. Non peer-reviewed papers, abstract shorter than three pages, lecture notes and thesis dissertations were excluded from the analysis. Particular attention was given to the customization principles and the mechanical and functional tests. For each topic, the main results from all relevant papers are reported and summarized herein. There were 75 papers that corresponded to the search criteria. These were grouped according to the following macro-topics: 16 focusing on scanning technologies and geometry acquisition; 14 on customization criteria; 19 on production techniques; 16 on mechanical testing, and 33 on functional testing. According to the present review, design and production of custom PD-AFOs are becoming increasingly feasible due to advancements in 3D scanning techniques and additive manufacturing. In general, custom PD-AFOs were shown to provide better comfort and improved spatio-temporal parameters with respect to standard solutions. However, no customization principle to adapt PD-AFO stiffness to the patient’s degree of ankle impairment or mechanical/functional demand has thus far been proposed.

## Background

Drop-foot is a severely disabling syndrome affecting the lower limb, generally associated with damage to or malfunction of the central or peripheral nervous system, such as peroneal nerve injury, or brain and spinal cord disorders. The term derives from the inability to dorsiflex the foot due to insufficiency of the main ankle dorsiflexor muscles, such as the tibialis anterior. This deficit is particularly critical in the swing phase of walking, resulting in higher risk of stumbling and falling. About 23% of patients with symptomatic herniated disc (incidence about 1% of EU population) and 20% of those affected by stroke (incidence about 0.1% of EU population) have been reported to suffer from foot drop [1, 2].

An Ankle-Foot Orthosis (AFO) is usually prescribed to compensate for the functional limitations due to the drop-foot condition. AFOs are meant to restore quasi-normal gait patterns in drop-foot patients by resisting the ankle joint moments in the swing phase of walking, thus reducing the risk of falling. While standard off-the-shelf AFOs are rather inexpensive (50–100 EUR), they have several limitations: 1) they are sold in limited sizes (e.g. small, medium and large); 2) they do not always match the patient’s foot and leg geometry; 3) they have fixed mechanical properties that cannot address patient-specific impairments or functional demand; 4) they do not address other foot morphological alterations, such as severely pronated feet.

Frequently, standard AFOs require further manual customization to include an orthotic insole. As reported in a recent review [3], AFO stiffness represents a key factor influencing the gait pattern of drop-foot patients, but no guidelines for AFO design customization have been established. While AFO stiffness is fundamental to sustain the foot in the swing phase, this mechanical parameter can affect the physiological ankle dorsiflexion and plantarflexion in the stance phase of walking.

**Fig. 1:**
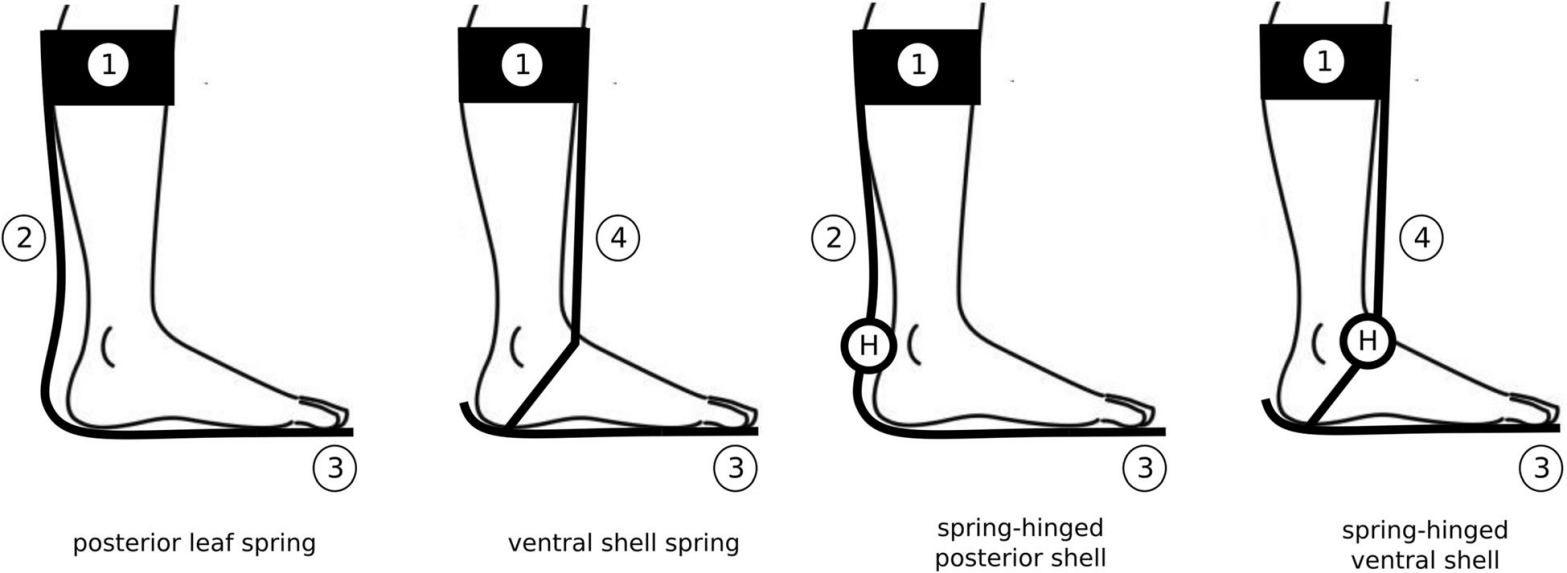
The four main types of PD-AFOs. Where: (1) is the calf strap; (2) is the calf shell; (3) is the foot plate, and (4) is the ventral shell. H is the variable or fixed-stiffness hinge joint connecting the foot plate to the calf or to the ventral shell

AFOs are largely classifiable in the two main groups of passive and active, which can be exploded in further subgroups as follows:

- Passive AFOs
  ○ Rigid or solid: characterized by stiff shells which prevent ankle movement in the three anatomical planes;
  ○ Dynamic: flexible in the sagittal plane, allow for some dorsi/plantarflexion movement. Flexibility can be provided by a deformable shell (non-articulated: e.g. posterior leaf spring or ventral shell spring as in Fig. 1) or via a fixed-stiffness hinge joint (articulated: spring-hinged posterior or ventral shell as in Fig. 1).
- Active AFOs: articulated and fitted with powered actuators. Flexion/extension movements at the ankle joint are actively assisted by the actuators.

This study is a literature review on the customization, production and testing of passive dynamic AFOs (PD-AFOs). Design, development and application of dynamic AFOs for patients with different degrees of drop-foot conditions are benefitting from the latest advancements in additive manufacturing. It is now possible to print an AFO shell in any shape and with a variety of materials using different 3D printing technologies. While traditional production techniques can also be used, additive manufacturing is fast becoming the new gold standard to produce custom orthotic devices with improved comfort and performance with respect to off-the-shelf solutions. While interest in this field is continuously growing [4], the process for the custom design, testing and evaluation of dynamic AFOs has not been established, and no standards have been published. Therefore, a large number of design principles, AFO materials and testing protocols have been reported. This critical review of the literature is aimed at collecting and reporting the major studies on custom PD-AFOs to date so as to highlight the major stepping stones in the development of a new generation of custom AFOs and to identify the major issues that still need to be overcome in this process.

## Material and methods

An extensive literature review was conducted between May and November 2021 on the Google Scholar online database. The following keywords were used, either alone or in combination, to find relevant papers for the present review: dynamic; AFO; ankle foot orthosis; custom; patient-specific orthotic; mechanical testing; functional evaluation; gait analysis; drop-foot; customization; 3D printing; additive manufacturing; comfort; design and finite element analysis (FEA). For the purposes of this review study, we defined PD-AFOs as orthoses characterized by significant flexibility of a posterior (i.e. posterior leaf spring) or anterior (i.e. ventral shell) support or fitted with a dynamic hinge joint with pre-compressed spring elements to control motion in the sagittal-plane (Fig. 1). Papers were not included in the review for the following reasons: not focusing on custom solutions (i.e. standard off-the-shelf AFOs); not focusing on passive dynamic AFOs (i.e. rigid or, active AFOs) or type not clearly defined. It should be pointed out that some authors used the term “dynamic” while referring to active AFOs — those with actuators. These papers, along with abstracts shorter than three pages, lecture notes, thesis dissertations and papers not published on peer-reviewed journals were also excluded from the review. The papers complying with the inclusion criteria were analyzed and grouped in five different macro-topics: a) scanning technologies and geometry acquisition; b) customization criteria; c) production techniques; d) mechanical testing, and e) functional evaluation.

## Results

A total of 242 papers were found. 75 of these complied with the inclusion criteria and were included in the review. Some of these papers covered more than one macro-topic specified in Material and Methods. The number of papers covering each topic follows:

- 16 papers addressed and collected patient geometry of the shank and foot;
- 14 papers reported AFO customization criteria other than those based on foot and leg morphology;
- 19 papers reported the production techniques;
- 16 papers investigated characterization of mechanical properties;
- 33 papers reported the functional evaluation of patients/subjects.

A summary of the main results from the literature review on each topic is summed up in the following subsections.

- Scanning technologies and geometry acquisition

Custom AFOs are traditionally modelled by hand by the orthotist via thermal molding on models of the patient’s foot and leg. Traditionally, the plaster model is obtained by filling the negative impression of the patient’s cast with liquid plaster. The custom AFO is then manufactured over the positive model. This process, however, is time-consuming and highly operator-dependent. Therefore, in the last 10 years, new technologies to obtain a 3D digital replica of the patient’s geometry have been used to create a solid model of the foot and leg: laser-based scanners [5–10] (6 out of 16 studies); structured-light scanners [11–13] (3/16); computer tomography [5, 14–16] (4/16); 3D coordinate digitizer to acquire landmark positions [17, 18] (2/16), and photogrammetry [19] (1/16). According to recent reviews [20, 21], 3D scanning, computer tomography and optical motion capture systems all represent valid and reliable alternatives to traditional casting methods to obtain a solid model of the patient’s foot and leg geometry.

b) Customization criteria

According to the present review, PD-AFOs are usually customized on the patient’s lower limb morphology. Few studies used a commercial customizable PD-AFO — the modular Intrepid Dynamic Exoskeletal Orthosis (IDEO) — featuring a posterior strut, the stiffness of which can be customized to the patient’s ankle ROM, the type and level of activities, body mass and load carriage requirements [22–25]. A similar modular design featuring a variable stiffness rod in relation to the patient’s degree of impairment was proposed [26]. However, no indications are provided on the weight and the direction (towards stiffer or more compliant) of each parameter on the strut rigidity. AFO stiffness optimization based on the minimization of knee angle and energy cost of walking was reported for children with cerebral palsy [27, 28]. A combination of the following parameters has also been used as input data to set the stiffness of the custom AFOs: the patient’s prior experience; visual observations of patient’s gait; body weight; muscle strength; severity of ankle deformity [29–33]. Only one study customized the AFO stiffness according to the natural ankle pseudo-stiffness [34]. The majority of the studies optimized the stiffness of the calf shell. Only one study reported the effect of footplate stiffness on ankle joint power in gait [35].

iii) Production techniques

Additive manufacturing is becoming widely used in orthopaedics, since it allows to obtain complex shaped devices made with a number of different materials [20]. The present review, in agreement with two recent studies [36, 37], has shown that most 3D-printed PD-AFOs are manufactured via Selective Laser Sintering (SLS) [5, 6, 8, 14, 15, 18, 25, 26, 38–42] and Fused Deposition Modeling (FDM) – also known as Fused Filament Fabrication (FFF) – [10, 15, 17, 43, 44]. SLS works with a high-power laser to sinter polymer powders, while FDM adds melted thermoplastic filaments in consecutive stratified layers to create the object. Stereolithography (SLA) [7, 11] and Multi Jet Fusion (MJF) [11] are less frequently used to produce custom AFOs. In SLA, a UV laser induces polymerization of a photopolymer to obtain the object; in MJF, a fusing agent is deposited on layers of heated powder where the particles are fused together.

iv) Mechanical testing

This section is reporting only studies related to the experimental analysis of custom-made PD-AFOs. Whenever the AFO type was not clearly defined as “dynamic”, it was decided to include only the manuscripts which reported the force/deformation properties, providing evidence of a dynamic behavior of the orthosis. Three review studies were found which reported stiffness values for a variety of AFOs — custom and off-the-shelf — and the testing methods [3, 45, 46]. Most of these studies investigated the stiffness properties in plantar-dorsiflexion in the range 20 deg plantar- to 30 deg dorsiflexion. Only one study assessed the AFO’s mechanical properties outside the sagittal plane [47].

Most studies assessed the stiffness properties of the strut component, i.e. the long, flexible part of the calf shell [17, 41, 47–51]. Fewer studies investigated the mechanical properties of other components, such as the foot plate [50], or isolated parts of the AFO [52]. Displacements during AFO deflection were assessed in two studies [49, 53], while only one study performed a fatigue test [44]. A few papers [17, 49, 52] reported the mechanical testing of dynamic AFOs which were customized on a healthy subject’s leg or on other geometrical models of the lower limb and not for drop-foot patients were included in this review. In general, the AFO foot plate is fixed, and bending moments/forces or displacements are applied to the calf shell, simulating ankle dorsiflexion. The reported bending stiffness of the strut, in terms of resistance to dorsiflexion moment, ranged between 0.12 and 8.9 N*m/deg across these studies [15, 17, 33, 41, 48–50]. The energy absorbed/released by custom AFOs during gait has been seldom addressed in the literature [29, 54].

Custom PD-AFOs have also been tested in-silico via FEA [17, 42, 48, 52–54]. Boundary conditions were generally consistent with those used for the experimental mechanical tests, when present. In addition to stiffness [17, 42], FEA allowed to estimate the maximum Von Mises stresses [52, 55] and displacements [53] of the analyzed AFOs. Only one study assessed the maximum Von Mises stress against the material yielding [52], and reported the safety factor of each component in simulated jogging and downhill walking tasks.

e) Functional evaluation

Table 1 sums up the outcome of the literature review in relation to the functional evaluation of custom dynamic AFOs. Thirty-three papers published from 1999 to 2021 were retrieved and found relevant to the topic. In terms of populations investigated, custom AFOs were used for post-stroke patients (*n* = 6) [11, 34, 57, 58, 62, 64], for generic drop-foot and muscles weakness (*n* = 13) [8, 24, 30–33, 39, 40, 44, 50, 51, 56, 59], for lower limb reconstruction (*n* = 4) [22, 23, 25, 60], for cerebral palsy (*n* = 4) [27, 28, 61, 66], for Charcot–Marie–Tooth (*n* = 1) [29], in children with hemiplegia (*n* = 2) [63, 65], and in normal/healthy subjects (*n* = 3) [7, 35, 43]. Posterior Leaf Spring (PLS) are the most common types of AFOs functionally evaluated and were compared to solid and hinged AFOs, and/or to shod/barefoot conditions. Carbon-fiber was found to be the most used material; plastic (nylon and polyamide) and thermoplastic (polypropylene and polyurethane) were also used due to their favorable manufacturing process and compatibility with current 3D printing technology. In terms of functional evaluation, gait analysis during walking at comfortable speed was by far the most common motor task investigated. Three studies reported on stair ascent/descent, and two studies reported on walking over an inclined ramp or treadmill. In one study, the AFOs were evaluated in a static balance test. Spatio-temporal parameters and lower limb joint kinematics and kinetics (mainly in the sagittal plane) were usually recorded and analyzed. Two studies also reported on surface EMG of the main lower limb muscles. Six studies reported on other qualitative scores such as comfort or ease of use (donning and removing). In terms of spatio-temporal parameters, while it is difficult to compare the functional outcome of PD-AFOs customized and produced for different populations with ankle weakness, 8 studies reported improved gait velocity and stride length in custom AFOs with respect to solid AFOs or shod/barefoot conditions. Due to the flexibility of the calf shell, custom PD-AFOs can absorb and release energy during walking. The two studies that assessed this parameter reported a reduction in the energy cost of walking while wearing the optimal stiffness AFOs with respect to other AFOs.

**Table 1:** Literature review with respect to the papers reporting on the functional evaluation of custom PD-AFOs. For each paper, when present, it is reported the AFO type(s), the customization criteria, the materials, the functional data/parameters, and the main outcome. Comfort assessment or other subjective scores are also reported

Authors/year	Population (size)	AFO type/ customization criteria	Material	Motor tasks	Functional parameters	Other scores	Main outcome
Waterval et al. 2021 [56]	unilateral plantar flexor weakness (9)	dorsal leaf spring AFO Spring leaf Stiffness customizable energy cost optimized (Ankle7, OttoBock)	carbon fiber	walking	spatio-temporal parameters GRFs hip, knee, ankle kinematics and kinetics		peak vertical GRF of the contralateral leg significantly reduced and symmetry improved (AFO vs. no AFO)
Waterval et al. 2021 & 2020 [32, 33]	calf muscle weakness (34)	dorsal leaf spring AFO Spring leaf Stiffness customizable (Ankle7, OttoBock)e	carbon fiber	walking	spatio-temporal parameters hip, knee, ankle kinematics and kinetics energy cost		reduction in energy cost (AFO optimized stiffness vs. non optimized)
Kerkum et al. 2021 [35]	healthy subjects (12)	dorsal leaf spring AFO Spring leaf Stiffness customizable (Ankle7, OttoBock)	carbon fiber	walking	Ankle-foot kinematics work and power		Total ankle-foot power increase with increasing footplate stiffness
Lin et al. 2021 [57]	post-stroke drop-foot (12)	1. energy-Storage 3D Printed AFO 2. anterior-support AFO	PLA + nylon+titanium thermoplastic	walking	spatio-temporal parameters pelvis, hip, knee, ankle kinematics (sagittal plane)	Evaluation of satisfaction (QUEST)	increased gait velocity and stride length (AFO1 vs. AFO2; AFO1 vs. barefoot) improved satisfaction (AFO1)
Meng et al. 2021 [58]	post-stroke drop-foot (15)	morphology	PA2200 Somos NeXt PA12	NA	NA	comfort weight feeling surface smoothness wearing issues cleaning issues	Somos NeXt scored better than one or more materials in comfort and surface smoothness
Vasiliauskaite, et al. 2020 [51]	child with unilateral drop-foot (1)	1. hinged AFO with adjustable ankle stiffness 2. posterior leaf spring stiffness tuned to achieve the orthotic goals	thermoplastic+metal polyamide-12	walking	spatio-temporal parameters hip, knee, ankle kinematics and kinetics	NA	Despite having the same ankle stiffness, AFO1 and AFO2 did not produce the same gait pattern
Chae et al. 2020 [59]	unilateral drop-foot (1)	morphology	polyurethane	walking stairs ascent/descent up&go	NA	Modified Emory Functional Ambulation Profile	improved mEFAP (AFO vs. no-AFO)
Esposito et al. 2020 [22]	unilateral lower limb reconstruction (12)	IDEO custom AFO (posterior leaf spring) Stiffness based body mass, load carriage requirements, and range of available pain-free motion	carbon fiber	walking	COP position COP velocity	NA	±3 deg in strut flexion/extension strut alignment does not significantly affect the foot-ankle roll-over shape radius
Liu et al. 2019 [11]	post-stroke drop-foot (12)	morphology	PA12	walking	spatio-temporal parameters hip, knee, ankle kinematics	NA	improved velocity and stride length (AFO vs.no-AFO)
Waterval et al. 2019 [50]	neuromuscular disorders and non-spastic calf muscle weakness (37)	dorsal leaf spring AFO (Carbon Ankle Seven, Ottobock, Duderstadt) adjustable stiffness	carbon fiber	walking	energy cost spatio-temporal parameters hip, knee, ankle kinematics and kinetics	NA	energy cost −20% (optimal AFO vs. no-AFO) energy cost − 10.7% (optimal AFO vs. non-optimal AFO)
Cha et al. 2017 [44]	unilateral drop-foot (1)	1. sock-like design with anterior opening and malleoli holes 2. rigid AFO	thermoplastic polyurethane	walking	spatio-temporal parameters ankle kinematics	Evaluation of satisfaction (QUEST)	insufficient ankle dorsiflexion in swing (AFO1 vs AFO2) better wearing properties and comfort (AFO1 vs AFO2))
Esposito et al. 2017 [23]	unilateral lower limb reconstruction (24)	IDEO custom AFO (posterior leaf spring) Stiffness based body mass, load carriage requirements, and range of available pain-free motion	carbon fiber	walking	spatio-temporal parameters hip, knee, ankle kinematics (sagittal plane)	NA	limited power capabilities at the ankle, and reduced compensatory strategies at the knee with respect to amputees
Arch & Reisman 2016 [34]	post-stroke (2)	custom AFOs Morphology-based, no shoe required	polycarbonate	walking	spatio-temporal parameters hip, knee, ankle kinematics and kinetics	NA	increased net peak plantarflexion moment and natural ankle pseudo-stiffness.
Whitehead et al. 2016 [60]	unilateral lower limb reconstruction (13) normal/healthy (13)	IDEO custom AFO (posterior leaf spring)	carbon fiber	stairs ascent/descent	spatio-temporal parameters hip, knee, ankle kinematics and kinetics (sagittal plane)	NA	stair ascent: greater bilateral hip power during pull-up and reduced ankle dorsiflexion and knee extensor moment (AFO vs. control)
Ranz et al. 2016 [38]	unilateral ankle muscle weakness (13)	IDEO custom AFO (posterior leaf spring) 3 bending axis positions	carbon fiber nylon 11 (strut)	walking	sEMG: soleus, gastrocnemius, tibialis ant., rectus fem., biceps fem., vastus med. and gluteus med. spatio-temporal parameters hip, knee, ankle kinematics and kinetics	NA	hip and knee moments were affected by bending axis position no difference in spatio-temporal parameters
Arch & Stanhope 2015 [43]	normal/healthy (2)	passive dynamic AFO (posterior leaf spring) AFO stiffness according to natural ankle pseudo-stiffness	not reported	walking	Ankle kinematics and moments (sagittal plane)	NA	
Haight at al. 2015 [25]	unilateral lower-limb reconstruction (12)	IDEO custom AFO (posterior leaf spring) variable stiffness based on ROM, activity level, types of activities, body mass, load carriage requirements	carbon fiber	treadmill uphill walking (10 deg slope)	spatio-temporal parameters hip, knee, ankle kinematics and kinetics	NA	AFOs stiffer than nominal increased knee joint flexion
Kerkum et al. 2015 & 2016, Meyns et al. 2020 [27, 28, 61]	children with cerebral palsy (15; bilateral 14)	ventral shell spring-hinged AFO (vAFO) variable stiffness/ROM hinge	pre-preg carbon fiber	waking	energy cost spatio-temporal parameters hip, knee, ankle kinematics and kinetics	NA	decreased net energy cost (vAFOs vs. no-AFO) no differences between vAFOs
Harper et al. 2014 [42]	unilateral ankle muscle weakness (10)	IDEO custom AFO (posterior leaf spring) clinically prescribed stiffness	carbon fiber nylon 11 (strut)	walking	spatio-temporal parameters hip, knee, ankle kinematics and kinetics	NA	no difference in kinematics/kinetics between the two materials (same AFO stiffness)
Esposito et al. 2014 [24]	unilateral ankle muscle weakness (13) healthy controls (13)	IDEO custom AFO (posterior leaf spring) variable stiffness based on ROM, activity level, types of activities, body mass, load carriage requirements	carbon fiber nylon 11 (strut)	walking	spatio-temporal parameters hip, knee, ankle kinematics and kinetics	NA	small differences in kinematics and kinetics (nominal stiffness vs. stiffer and more compliant)
Dufek et al. 2014 [29]	Charcot–Marie–Tooth patients (bilateral 8)	posterior leaf spring AFO stiffness customization based on prior experience, visual observations of patient’s gait, weight and muscle strength, and amount of ankle deformity	carbon-fiber composite	walking	spatio-temporal parameters hip, knee, ankle kinematics and kinetics	NA	increased walking speed and stride length (custom AFO vs. no-AFO) AFO energy storage 9.6 ± 6.6 J/kg
Creylman et al. 2013 [8]	unilateral drop foot (8)	morphology-based posterior leaf spring/shell	nylon 12 (AFO1) polypropylene (AFO2)	walking	spatio-temporal parameters hip, knee, ankle kinematics (sagittal plane)	NA	improved spatial temporal gait parameters and ankle kinematics (AFO1 & AF2 vs. no-AFO)
Mavroidis et al. 2011 [7]	normal/healthy (1)	morphology-based posterior leaf spring/shell (based on Type C-90 Superior Posterior Leaf Spring, AliMed)	polypropylene (AFO1, standard) Accura SI 40 (AFO2) Somos 9121 (AFO3)	walking	spatio-temporal parameters ankle kinematics and kinetics (sagittal plane)	comfort	comparable functional outcome to standard AFO and better comfort (AFO2 and AFO3 vs AFO1)
Lewallen et al. 2010 [62]	post-stroke drop-foot (13)	solid AFO vs. hinged vs. posterior leaf spring	thermoplastics	walking walking up/down 10 deg ramp	spatio-temporal parameters	NA	significantly reduced walking speed and stride length (solid AFO vs. all AFOs and no-AFO) only one subject preferred solid AFO over the other AFOs
Bartonek et al. 2007 [31]	children with bilateral ankle muscle weakness (11 AFO; 6 KAFO)	morphology-based posterior leaf spring patient’s level of functional ambulation and body weight	pre-preg carbon-fiber	walking	spatio-temporal parameters hip, knee, ankle kinematics (sagittal plane)	frequency of use gait standing function changes walking velocity acceptance ease of putting on and removing	for most children, improved ankle plantarflexion moment (*p* < 0.001), ankle positive work (*p* < 0.001), and stride length (*p* < 0.001) (custom AFO vs. rigid shell thermoplastic AFO)
Bartonek et al. 2007 [30]	children with bilateral ankle muscle weakness (2 AFO; 1 KAFO)	morphology-based posterior leaf spring patient’s level of functional ambulation and body weight	pre-preg carbon-fiber	walking	spatio-temporal parameters hip, knee, ankle kinematics (sagittal plane)	frequency of use gait standing function changes walking velocity acceptance ease of putting on and removing	increased stride length (2/2; custom AFO vs. rigid shell thermoplastic AFO) increased walking speed (1/2) perceived improved gait
Desloovere et al. 2006 [63]	children with hemiplegia (15)	flexible posterior leaf-springs (PLS) Dual Carbon Fibre Spring AFO (CFO) clinical examination and gait analysis	thermoplastic thermoplastic & carbon and kevlar fibres pre-impregnated with epoxy (strut)	walking	spatio-temporal parameters hip, knee, ankle kinematics	NA	increased walking speed and stride length (PLS vs. no-AFO) larger ankle ROM and ankle velocity during push-off increased plantar flexion moment and power generation at pre-swing (CFO vs. PLS; *p* < 0.01).
Gök et al. 2003 [64]	hemiparetic stroke patients (12)	1. Seattle-type polypropylene AFO 2. metallic AFO	polypropylene metal	walking	spatio-temporal parameters hip, knee, ankle kinematics	NA	increased walking speed (AFO2 vs AFO1 vs. no-AFO) increased stride length (AFO1 vs. no-AFO; AFO2 vs. no-AFO)
Sienko Thomas et al. 2002 [65]	children spastic hemi-plegia (19)	morphology-based 1. hinged AFO 2. posterior leaf spring (PLS) 3. solid AFO	thermoplastic	walking stairs ascent/descent	spatio-temporal parameters pelvis, hip, knee, ankle kinematics (sagittal plane)	Pediatric Evaluation of Disability Inventory (PEDI)	reduced ankle plantarflexion (AFOs vs. barefoot)
Burtner et al. 1999 [66]	children with spastic diplegic cerebral palsy (4, and 4 healthy control)	1. solid AFO 2. dynamic (spiral) AFO	Polypropylene graphite	static balance test	sEMG: gastrocnemius, tibialis ant., hamstrings, quadriceps, paraspinals, abdominals. hip, knee, ankle kinematics (sagittal plane)	NA	decreased activation of gastrocnemius, disorganized muscle-response patterns, decreased use of ankle strategies, increased knee joint angular velocity (AFO1 vs. AFO2 and AFO1 vs no-AFO) without AFOs or with dynamic AFOs.

## Discussion

The present review is aimed at investigating the current literature on the state-of-the-art of custom PD-AFOs design, production and testing. Although thermal molding of AFOs on solid models of the patients’ legs is the most used production method worldwide, 3D scanning techniques and additive manufacturing are becoming increasingly used in the production process of custom PD-AFOs. The following sections address the critical analysis of the literature on the macro-topics used to classify the studies.

The use of modern 3D scanning technologies allows for fast and accurate digitization of the patient’s foot and leg. Data can be stored and shared in various 3D file formats (e.g. STL, OBJ, etc.) which can be easily edited with several commercial (e.g. SolidWorks, Blender) or proprietary software. Some file formats are fully compatible with 3D printers (e.g. STL), thus the timing from geometry acquisition to AFO production is significantly reduced. Moreover, the same 3D file can be used and revised later without the need for a new acquisition of the patient’s geometry — in cases of wearing out, breakage or changes in the mechanical requirements. The dematerialization of the foot and leg 3D acquisition allows to share the geometry with the production facility, which can be remotely located with respect to the patient’s location. According to the present review, laser-based and structured-light scanners are the most common technologies for geometry acquisition. While the cost of high-quality 3D scanners and the expertise required to process the 3D files is limiting the spread of this technology, the development of low-cost 3D scanning solutions [13, 21, 67] is allowing more orthotic centers and research groups to advance the current methodology for geometrical reproduction from the traditional casting techniques.

The majority of the AFOs were customized according to morphological parameters only, regardless of body weight and/or functional requirement. Some studies reported that the AFO’s stiffness was customized on the patient’s body mass, load carriage requirements and/or range of available pain-free motion; however, the customization process was not sufficiently explained [22–25, 29–31, 34]. The IDEO was one of the most used custom AFOs across all studies [22–25, 39, 40, 60]. Although the IDEO can be customized to each patient and modulated according to changes in strength and functional ability [68], the relationship between the patient’s clinical deficit and functional requirements and the AFO’s mechanical properties and design features has not been standardized and reported to date.

With respect to AFO production, the growing demand for customized solutions is paving the way for additive manufacturing in the healthcare industry. 3D printers allow production of orthotic devices with complex shapes and have been successfully used to manufacture AFOs using a variety of materials, mostly polymers and composites. The combination of different materials in the same orthotic device is also possible. SLS was the most used technology to produce custom AFOs, as this allows several items to be produced simultaneously and has a lower environmental impact than FDM [69]. Although SLA and FDM are the most cost-effective solutions, SLS guarantees the highest accuracy and the fastest printing time [70].

Custom AFO stiffness was evaluated via mechanical tests simulating ankle flexion in gait. The bending stiffness, in terms of resistance to dorsiflexion moment, was significantly variable across studies as a consequence of the chosen design, material and thickness of the calf shell. Although standard testing methods to assess AFO stiffness under realistic biomechanical conditions have been proposed [71–73], most research groups developed custom setups and loading/displacement parameters. This made it difficult to compare the mechanical properties of AFOs with respect to materials and designs. Despite the importance of foot plate flexibility with respect to forefoot biomechanics in late stance, its mechanical properties and their effects on lower limb kinematics have not been sufficiently investigated to date. FEA allows to identify critical regions in terms of stress and strain under physiological loading conditions and to redesign high-stress regions exceeding the material yielding. This is particularly critical for dynamic AFOs with complex shapes subjected to large deformations. In addition, FEA is useful to minimize production costs by assessing different design solutions and materials before manufacturing. In terms of functional evaluation, the present review has revealed generally positive outcomes of custom AFOs with respect to the no-AFO condition and off-the-shelf/solid AFOs. While spatio-temporal, lower limb kinematic and kinetic parameters were frequently reported, subjective scores — such as comfort, walking confidence and ease of donning — were seldom implemented. Custom solutions scored better than standard/solid AFOs for comfort and satisfaction [7, 44, 57, 62]. It should be highlighted that the custom solutions were assessed against either barefoot or shod conditions. These two control conditions are biomechanically different, as the shoe’s weight significantly increases the plantarflexion moment at the ankle in the swing phase. Walking is the motor task most reported in the functional evaluation of AFOs. Few studies reported on other tasks, such as stair ascending/descending and ramp climbing. There is a lack of information on the biomechanical interaction between AFO and foot/leg for other major activities of daily living.

The present review should be interpreted with respect to some limitations. Since this study was meant to be a comprehensive literature review of the state of the art on PD-AFOs and not a systematic review, a research question was not formulated to compare the main outcomes across studies. The literature review was conducted on the Google Scholar database only. In addition, several studies did not clarify the AFO type — i.e. static/dynamic, standard/custom — therefore their classification was rather difficult, and these were excluded from the review.

## Conclusions

According to the present review, custom PD-AFOs are becoming increasingly feasible due to advancements in 3D scanning techniques and in additive manufacturing. In general, custom PD-AFOs provide better comfort and more physiological spatio-temporal parameters than standard off-the-shelf solutions. However, no clear customization principles to customize PD-AFO stiffness with respect to the patient-specific degree of impairment or mechanical and functional request have thus far been proposed and reported. Healthcare providers and clinicians should agree and inform on which clinical, morphological or functional parameters are critical to the PD-AFO customization process. Scoring systems to quantify the relevant parameters should also be formulated to obtain a global score which can be associated to the most appropriate AFO stiffness. A standard testing method to measure AFO stiffness is necessary to allow quantitative comparison between PD-AFO types and materials.

## Data Availability

Data sharing not applicable to this article as no datasets were generated or analysed during the current study.

## Abbreviations

AFO: Ankle-Foot Orthosis
PD-AFO: Passive Dynamic Ankle Foot Orthosis
FEA: Finite Element Analysis
SLS: Selective-Laser Sintering
FDM: Fused Deposition Modeling
SLA: Stereo Lithography
MJF: Multi-Jet Fusion
FFF: Fused Filament Fabrication
PLS: Posterior Leaf Spring
IDEO: Intrepid Dynamic Exoskeletal Orthosis

## References

[1] Ma J, He Y, Wang A, Wang W, Xi Y, Yu J, Ye X (2018). Risk factors analysis for foot drop associated with lumbar disc herniation: an analysis of 236 patients. World Neurosurg.

[2] Burridge JH, Taylor PN, Hagan SA, Wood DE, Swain ID (1997). The effects of common peroneal stimulation on the effort and speed of walking: a randomized controlled trial with chronic hemiplegic patients. Clin Rehabil.

[3] Totah D, Menon M, Jones-Hershinow C, Barton K, Gates DH (2019). The impact of ankle-foot orthosis stiffness on gait: a systematic literature review. Gait Posture.

[4] Wixted CM, Peterson JR, Kadakia RJ, Adams SB (2021). Three-dimensional printing in Orthopaedic surgery: current applications and future developments. J Am Acad Orthop Surg Glob Res Rev.

[5] Milusheva SM, Tosheva EY, Hieu LC, Kouzmanov LV, Zlatov N, Toshev YE (2006). Personalised Ankle-Foot Orthoses Design based on Reverse Engineering.

[6] Pallari JHP, Dalgarno KW, Munguia J, Muraru L, Peeraer L, Telfer S (2010). Design and additive fabrication of foot and ankle-foot orthoses.

[7] Mavroidis C, Ranky RG, Sivak ML, Patritti BL, DiPisa J, Caddle A, Gilhooly K, Govoni L, Sivak S, Lancia M, Drillio R, Bonato P (2011). Patient specific ankle-foot orthoses using rapid prototyping. J Neuroeng Rehabil.

[8] Creylman V, Muraru L, Pallari J, Vertommen H, Peeraer L (2013). Gait assessment during the initial fitting of customized selective laser sintering ankle foot orthoses in subjects with drop foot. Prosthetics Orthot Int.

[9] Roberts A, Wales J, Smith H, Sampson CJ, Jones P, James M (2016). A randomised controlled trial of laser scanning and casting for the construction of ankle-foot orthoses. Prosthetics Orthot Int.

[10] Shih A, Park DW, Yang YY, Chisena R, Wu D (2017). Cloud-based design and additive manufacturing of custom orthoses. Procedia CIRP.

[11] Liu Z, Zhang P, Yan M, Xie Y, Huang G (2019). Additive manufacturing of specific ankle-foot orthoses for persons after stroke: a preliminary study based on gait analysis data. Math Biosci Eng.

[12] Pandey R, Singh R (2021). Experimental Analysis of Various Materials on Custom-Fit Ankle Foot Orthosis. J Phys Conf Ser.

[13] Dombroski CE, Balsdon MER, Froats A (2014). The use of a low cost 3D scanning and printing tool in the manufacture of custom-made foot orthoses: a preliminary study. BMC Res Notes.

[14] Faustini MC, Neptune RR, Crawford RH, Stanhope SJ (2008). Manufacture of passive dynamic ankle-foot orthoses using selective laser sintering. IEEE Trans Biomed Eng.

[15] Walbran M, Turner K, McDaid AJ (2016). Customized 3D printed ankle-foot orthosis with adaptable carbon fibre composite spring joint. Cogent Eng.

[16] Alam M, Choudhury IA, Bin MA, Hussain S (2015). Computer aided design and fabrication of a custom articulated ankle foot orthosis. J Mech Med Biol.

[17] Schrank ES, Hitch L, Wallace K, Moore R, Stanhope SJ (2013). Assessment of a virtual functional prototyping process for the rapid manufacture of passive-dynamic ankle-foot orthoses. J Biomech Eng.

[18] Schrank ES, Stanhope SJ (2011). Dimensional accuracy of ankle-foot orthoses constructed by rapid customization and manufacturing framework. J Rehabil Res Dev.

[19] Dal Maso A, Cosmi F (2019). 3D-printed ankle-foot orthosis: a design method. Mater Today Proc.

[20] Barrios-Muriel J, Romero-Sánchez F, Alonso-Sánchez FJ, Salgado DR (2020). Advances in orthotic and prosthetic manufacturing: a technology review. Materials (Basel).

[21] Farhan M, Wang JZ, Bray P, Burns J, Cheng TL (2021). Comparison of 3D scanning versus traditional methods of capturing foot and ankle morphology for the fabrication of orthoses: a systematic review. J Foot Ankle Res.

[22] Esposito ER, Ruble MD, Ikeda AJ, Wilken JM (2020). The effect of custom carbon ankle-foot orthosis alignment on roll-over shape and center of pressure velocity.

[23] Russell Esposito E, Stinner DJ, Fergason JR, Wilken JM (2017). Gait biomechanics following lower extremity trauma: amputation vs. reconstruction. Gait Posture.

[24] Russell Esposito E, Blanck RV, Harper NG, Hsu JR, Wilken JM (2014). How does ankle-foot orthosis stiffness affect gait in patients with lower limb salvage?. Clin Orthop Relat Res.

[25] Haight DJ, Russell Esposito E, Wilken JM (2015). Biomechanics of uphill walking using custom ankle-foot orthoses of three different stiffnesses. Gait Posture.

[26] Deckers JP, Vermandel M, Geldhof J, Vasiliauskaite E, Forward M, Plasschaert F (2018). Development and clinical evaluation of laser-sintered ankle foot orthoses. Plast Rubber Compos.

[27] Kerkum YL, Harlaar J, Buizer AI, van den Noort JC, Becher JG, Brehm M-A (2016). An individual approach for optimizing ankle-foot orthoses to improve mobility in children with spastic cerebral palsy walking with excessive knee flexion. Gait Posture.

[28] Meyns P, Kerkum YL, Brehm MA, Becher JG, Buizer AI, Harlaar J (2020). Ankle foot orthoses in cerebral palsy: effects of ankle stiffness on trunk kinematics, gait stability and energy cost of walking. Eur J Paediatr Neurol.

[29] Dufek JS, Neumann ES, Hawkins MC, O’Toole B (2014). Functional and dynamic response characteristics of a custom composite ankle foot orthosis for Charcot-Marie-tooth patients. Gait Posture.

[30] Bartonek Å, Eriksson M, Gutierrez-Farewik EM (2007). A new carbon fibre spring orthosis for children with plantarflexor weakness. Gait Posture.

[31] Bartonek Å, Eriksson M, Gutierrez-Farewik EM (2007). Effects of carbon fibre spring orthoses on gait in ambulatory children with motor disorders and plantarflexor weakness. Dev Med Child Neurol.

[32] Waterval NFJ, Brehm M-A, Altmann VC, Koopman FS, Den Boer JJ, Harlaar J (2020). Stiffness-optimized ankle-foot orthoses improve walking energy cost compared to conventional orthoses in neuromuscular disorders: a prospective uncontrolled intervention study. IEEE Trans Neural Syst Rehabil Eng.

[33] Waterval NFJ, Brehm M-A, Harlaar J, Nollet F (2021). Individual stiffness optimization of dorsal leaf spring ankle–foot orthoses in people with calf muscle weakness is superior to standard bodyweight-based recommendations. J Neuroeng Rehabil.

[34] Arch ES, Reisman DS (2016). Passive-dynamic ankle-foot orthoses with personalized bending stiffness can enhance net Plantarflexor function for individuals Poststroke. J Prosthetics Orthot.

[35] Kerkum YL, Philippart W, Houdijk H (2021). The effects of footplate stiffness on push-off power when walking with posterior leaf spring ankle-foot orthoses. Clin Biomech.

[36] Wojciechowski E, Chang AY, Balassone D, Ford J, Cheng TL, Little D, Menezes MP, Hogan S, Burns J (2019). Feasibility of designing, manufacturing and delivering 3D printed ankle-foot orthoses: a systematic review. J Foot Ankle Res.

[37] Shahar FS, Hameed Sultan MT, Shah AUM, Azrie Safri SN (2020). A comparative analysis between conventional manufacturing and additive manufacturing of ankle-foot orthosis. Appl Sci Eng Prog.

[38] Telfer S, Pallari J, Munguia J, Dalgarno K, McGeough M, Woodburn J (2012). Embracing additive manufacture: implications for foot and ankle orthosis design. BMC Musculoskelet Disord.

[39] Harper NG, Russell EM, Wilken JM, Neptune RR (2014). Selective laser sintered versus carbon fiber passive-dynamic ankle-foot orthoses: a comparison of patient walking performance. J Biomech Eng.

[40] Ranz EC, Russell Esposito E, Wilken JM, Neptune RR (2016). The influence of passive-dynamic ankle-foot orthosis bending axis location on gait performance in individuals with lower-limb impairments. Clin Biomech.

[41] Takahashi KZ, Stanhope SJ (2010). Estimates of stiffness for ankle-foot orthoses are sensitive to loading conditions. J Prosthetics Orthot.

[42] Faustini MC, Neptune RR, Crawford RH, Stanhope SJ. Selective laser sintering of passive dynamic ankle-foot orthoses. 17th Solid Free. Fabr. Symp. SFF 2006; 2006. p. 124–9. 10.1109/TBME.2007.912638 18270017

[43] Arch ES, Stanhope SJ (2015). Passive-dynamic ankle–foot orthoses substitute for ankle strength while causing adaptive gait strategies: a feasibility study. Ann Biomed Eng.

[44] Cha YH, Lee KH, Ryu HJ, Joo IW, Seo A, Kim DH, Kim SJ (2017). Ankle-foot orthosis made by 3D printing technique and automated design software. Appl Bionics Biomech.

[45] Kobayashi T, Leung AKL, Hutchins SW (2011). Techniques to measure rigidity of ankle-foot orthosis: a review. J Rehabil Res Dev.

[46] Ielapi A, Forward M, De Beule M (2019). Computational and experimental evaluation of the mechanical properties of ankle foot orthoses: a literature review. Prosthetics Orthot Int.

[47] Cappa P, Patanè F, Di Rosa G (2005). A continuous loading apparatus for measuring three-dimensional stiffness of ankle-foot orthoses. J Biomech Eng.

[48] Ielapi A, Lammens N, Van Paepegem W, Forward M, Deckers JP, Vermandel M (2019). A validated computational framework to evaluate the stiffness of 3D printed ankle foot orthoses. Comput Methods Biomech Biomed Engin.

[49] Wach A, McGrady L, Wang M, Silver-Thorn B. Assessment of mechanical characteristics of ankle-foot orthoses. J Biomech Eng. 2018;140(7). 10.1115/1.4039816. 10.1115/1.4039816 29715361

[50] Waterval NFJ, Nollet F, Harlaar J, Brehm MA (2019). Modifying ankle foot orthosis stiffness in patients with calf muscle weakness: gait responses on group and individual level. J Neuroeng Rehabil.

[51] Vasiliauskaite E, Van Paepegem W, Deckers JP, Vermandel M, Forward M, Plasschaert F (2020). Additive manufacturing of ankle-foot orthosis with predefined ankle stiffness - a case report. J Prosthetics Orthot.

[52] Li W, Lemaire ED, Baddour N (2020). Design and evaluation of a modularized ankle-foot orthosis with quick release mechanism. 2020 42nd Annu. Int. Conf. IEEE Eng. Med. Biol. Soc., IEEE.

[53] Surmen HK, Akalan NE, Fetvaci MC, Arslan YZ (2018). A novel dorsal trimline approach for passive-dynamic ankle-foot orthoses. Strojniški Vestnik-J Mech Eng.

[54] Zou D, He T, Dailey M, Smith KE, Silva MJ, Sinacore DR, Mueller MJ, Hastings MK (2014). Experimental and computational analysis of composite ankle-foot orthosis. J Rehabil Res Dev.

[55] Surmen HK, Akalan NE, Arslan YZ (2018). Design, Manufacture, and Selection of Ankle-Foot-Orthoses. Encycl. Inf. Sci. Technol. Fourth Ed., IGI Global.

[56] Waterval NFJ, Brehm MA, Harlaar J, Nollet F (2021). Energy cost optimized dorsal leaf ankle-foot-orthoses reduce impact forces on the contralateral leg in people with unilateral plantar flexor weakness. Gait Posture.

[57] Lin CC, Yeh CH, Tsai YC, Kuo LC, Hsu HY, Chuang PH, Chang K, Su FC (2021). Evidence-based customized ankle-foot orthosis with energy storage. J Med Biol Eng.

[58] Meng X, Ren M, Zhuang Y, Qu Y, Jiang L, Li Z (2021). Application experience and patient feedback analysis of 3D printed AFO with different materials: a random crossover study. Biomed Res Int.

[59] Chae DS, Kim DH, Kang KY, Kim DY, Park SW, Park SJ, Kim JH (2020). The functional effect of 3D-printing individualized orthosis for patients with peripheral nerve injuries: three case reports. Medicine (Baltimore).

[60] Aldridge Whitehead JM, Russell Esposito E, Wilken JM (2016). Stair ascent and descent biomechanical adaptations while using a custom ankle–foot orthosis. J Biomech.

[61] Kerkum YL, Buizer AI, Van Den Noort JC, Becher JG, Harlaar J, Brehm MA (2015). The effects of varying ankle foot orthosis stiffness on gait in children with spastic cerebral palsy who walk with excessive knee flexion. PLoS One.

[62] Lewallen J, Miedaner J, Amyx S, Sherman J (2010). Effect of three styles of custom ankle foot orthoses on the gait of stroke patients while walking on level and inclined surfaces. J Prosthetics Orthot.

[63] Desloovere K, Molenaers G, Van Gestel L, Huenaerts C, Van Campenhout A, Callewaert B (2006). How can push-off be preserved during use of an ankle foot orthosis in children with hemiplegia? A prospective controlled study. Gait Posture.

[64] Gök H, Küçükdeveci A, Altinkaynak H, Yavuzer G, Ergin S (2003). Effects of ankle-foot orthoses on hemiparetic gait. Clin Rehabil.

[65] Sienko Thomas S, Buckon CE, Jakobson-Huston S, Sussman MD, Aiona MD (2002). Stair locomotion in children with spastic hemiplegia: the impact of three different ankle foot orthosis (AFOs) configurations. Gait Posture.

[66] Burtner PA, Woollacott MH, Qualls C (1999). Stance balance control with orthoses in a group of children with spastic cerebral palsy. Dev Med Child Neurol.

[67] Rogati G, Leardini A, Ortolani M, Caravaggi P (2019). Validation of a novel Kinect-based device for 3D scanning of the foot plantar surface in weight-bearing. J Foot Ankle Res.

[68] Highsmith MJ, Nelson LM, Carbone NT, Klenow TD, Kahle JT, Hill OT, Maikos JT, Kartel MS, Randolph BJ (2016). Outcomes associated with the intrepid dynamic exoskeletal orthosis (IDEO): a systematic review of the literature. Mil Med.

[69] Tagliaferri V, Trovalusci F, Guarino S, Venettacci S (2019). Environmental and economic analysis of FDM, SLS and MJF additive manufacturing technologies. Materials (Basel).

[70] Kafle A, Luis E, Silwal R, Pan HM, Shrestha PL, Bastola AK (2021). 3D/4D printing of polymers: fused deposition modelling (FDM), selective laser sintering (SLS), and stereolithography (SLA). Polymers (Basel).

[71] Bregman DJJ, Rozumalski A, Koops D, de Groot V, Schwartz M, Harlaar J (2009). A new method for evaluating ankle foot orthosis characteristics: BRUCE. Gait Posture.

[72] Totah D, Menon M, Gates DH, Barton K (2021). Design and evaluation of the SMApp: a stiffness measurement apparatus for ankle–foot orthoses. Mechatronics.

[73] Lai H-J, Yu C-H, Kao H-C, Chen W-C, Chou C-W, Cheng C-K (2010). Ankle–foot simulator development for testing ankle–foot orthoses. Med Eng Phys.

